# Hints to Young Practitioners—No. V

**Published:** 1872-11

**Authors:** 

**Affiliations:** Savannah, Georgia


### HINTS TO YOUNG PRACTITIONERS—NO. V.

BY SENEX.

"*Propter uterum est mulier.*"

To relieve the multitudinous forms of uterine trouble will constitute no small part of your practice. You read in books about procidentias, and versions, and flexions—about pelvic cellulitis, aud perimetritis, and endometritis, etc., etc. Be not dismayed. There is a large field for study and observation, but the principle is simple. The tendency of the times is too much gynæcology. A meddlesome midwifery is a bad midwifery, and the same may be said of uterine therapeutics. Many a functional trouble is fixed by the very means resorted to for its removal.

Dr. Laycock, in a work styled "Mind, Brain, and the Correlation of Consciousness," says: "When attention is directed to any portion of the body, innervation and circulation are excited locally, and functional activity of the organ is developed. Morbid anxiety about an organ invites disease."

Formerly, when a woman had backache, leucorrhœa, etc., we gave her muriated tincture iron, and directed an astringent injection, with cold bath and plenty of out-door exercise; now, such a case is subjected to a speculum examination. Something wrong is sure to be found about the cervix; caustic is applied, an ulcer is made, and then there is no end to the doctoring. So great has it become the fashion to resort to the speculum, that the Doctor will be considered ignorant of his business if he does not require an ocular inspection; and it calls for no small share of moral courage and honesty to stem the torrent of a vitiated public sentiment. Doubtless there are conditions when the speculum is valuable, both as a means of diagnosis and of applying topical treatment; but a great majority of the cases in which we are consulted do not require it. Dr. Robert Lee *(clarum et venerabile nomen)*, of England, denounces with much severity the unnecessary and dishonest use of this instrument. Of those who are base enough to subject their patients to needless examinations from mercenary motives, nothing too severe can be said; they disgrace a noble profession.

On this subject, let us hear what the admirable Sir Charles West says: "At the same time, however, that I hold the speculum to be in many cases of most essential service, I think that our endeavor should be to ascertain the minimum of frequency with which its employment is necessary. This is to be done by soberly and honestly trying to test the value of the information which we derive from it, and learning to discriminate between those appearances which the speculum discloses that are of moment and such as are of no importance."

Uterine sounds, sponge tents, etc., require much caution in their use. Serious consequences have often followed them, even in the most skillful hands. I knew an eminent physician induce miscarriage and serious peril by introducing a Simpson's sound, when a little patient waiting and a few doses of "tincture of time" (as old Gooch used to call the masterly inactivity policy), would have determined his diagnosis, and saved his patient from risk and grievous disappointment. "*Sunt plures medici qui œgros obid interimunt, quia nesciunt ipsi quiescere.*"

In regard to displacement, flexions, etc., the question of pessaries will arise. Nine times in ten, I believe the whole tribe of them to be unnecessary. Women wear them and don't wear them, with about equal results. A good rule is to try one, and if your patient is sensible of comfort from it, well; if not, I would prefer an extermінal abdominal support.

I had quite a number of lady friends who made their annual pilgrimages to Philadelphia to get *fixed up* by Dr. Hodge. We all respect this good and able teacher, but he certainly rode the pessary hobby hard. In very many of these cases, if the general health be cared for, the local ailments will disappear. There are many females—especially those who can afford to do so—who occupy themselves in nursing their complaints; and these will by no means be satisfied unless their supposed maladies are dignified with a Greek name, and the Doctor *does something* for them. This is an evil that at present there appears to be no cure for. We must amuse them for their own sakes, as well as ours, but let us be quite sure that we do no harm. The time will come when it will be deemed as wise not to do as to do, but that seems far distant.

We live in an age of marvelous progress, and woe to him who lags. "Be sure you are right, then go ahead," was a wise saying of the famous Davy Crockett. Shade of William Hunter! if permitted again to "revisit the glimpses of the moon," what would'st thou think of spaying a woman to cure her hysteria!! Is it any wonder that the public flies for refuge to the arms of homœopathy? Better King Log than King Stork.

SAVANNAH, GEORGIA, November 20th, 1872.

